# *Echinococcus multilocularis* in Estonia

**DOI:** 10.3201/eid1112.050339

**Published:** 2005-12

**Authors:** Epp Moks, Urmas Saarma, Harri Valdmann

**Affiliations:** * University of Tartu, Tartu, Estonia; † Estonian Biocentre, Tartu, Estonia

**Keywords:** Echinococcus multilocularis, Europe, red fox (Vulpes vulpes), letter

**To the Editor:** Alveolar echinococcosis (AE) caused by *Echinococcus multilocularis* is one of the most important emerging zoonosis in Europe. The fatality rate is >90% in untreated patients (*1*). In Europe, the distribution range of the zoonotic tapeworm *E. multilocularis* has expanded over the last few decades, and the parasite attracts increasing awareness as a public health issue (*2**–**5*). In 2003, AE was added to the list of zoonoses to be monitored in the member states of the European Union, according to Directive 2003/99/EC.

This is the first report of *E. multilocularis* in Estonia, which extends its northern distribution in Europe. Results of examinations of 17 red foxes shot in the eastern (Võnnu and Räpina) and western (Hiiumaa) districts of Estonia from February to December 2003 were included in this study. We examined the intestinal tracts by the sedimentation and counting technique as described (*1*). *Echinococcus* adult stages were found in 5 foxes (29.4%). Two foxes, infected with 3 and 5 adult worms, were from the Räpina district; 2 foxes, infected with 66 and 133 worms, were from the Võnnu district; and 1 fox, infected with the highest number of worms (927), was from the Hiiumaa District. The worms were retrieved, counted, washed, and stored in 90% ethanol until DNA purification. The parasites were identified as *E. multilocularis*, based on the most important morphometric parameters of adult stages (length of worms, number of proglottids, terminal proglottids in percentage of total worm length, position of genital pore, and form of uterus) (*2*).

To confirm the taxonomic status of the worms, polymerase chain reaction (PCR) was conducted, followed by restriction fragment length polymorphism (RFLP) analysis and direct sequencing of a portion of the NADH dehydrogenase subunit I (ND1) gene of the mtDNA. A total of 6 specimens of *E. multilocularis* were used for genetic analysis. Total genomic DNA was extracted with the High Pure PCR Template Preparation Kit (Roche Molecular Biochemicals, Mannheim, Germany) according to manufacturer's instructions. PCR-RFLP was performed as described by Gonzalez et al. (*6*). The RFLP pattern of *E. multilocularis* isolates differed from that of *E. granulosus*. Diagnostic cleavage at the locus Eg9 of *E. multilocularis* with the enzyme *CfoI* is able to distinguish *E. multilocularis* and its closest relative *E. granulosus* (Figure, lanes 3 and 4 vs. lane 10). All 6 specimens of *E. multilocularis* produced identical results. A 426-bp fragment of the mitochondrial ND1 gene was amplified with the primers NDfor2-AGTTTCGTAAGGGTCCTAATA and NDrev2-CCCACTAACTAACTCCCTTTC using the BD Advantage 2 PCR Kit (Becton Dickinson Biosciences, Franklin Lakes, NJ, USA) as described (*7*). DNA cycle sequencing was performed by using the DYEnamic ET Terminator Cycle Sequencing Kit (Amersham Pharmacia Biotech, Piscataway, NJ, USA). Sequences were resolved on an ABI PRISM 377 automated DNA sequencer (Applied Biosystems, Foster City, CA, USA).

**Figure:**
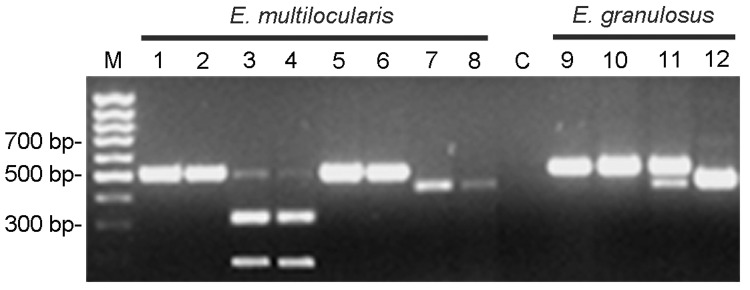
Diagnostic polymerase chain reaction (PCR) restriction fragment length polymorphism analysis for *Echinococcus multilocularis* (lanes 1–8, 2 specimens in parallel) and *E. granulosus* (lanes 9–12, 1 specimen). Lane M: Gene Ruler 100-bp DNA ladder; lane C: negative control without DNA; lanes 1 and 2: amplification of *E. multilocularis* DNA with Eg9 PCR; lanes 3 and 4: amplification of *E. multilocularis* DNA with Eg9 PCR, followed by cleavage with enzyme *CfoI*; lanes 5 and 6: amplification of *E. multilocularis* DNA with Eg9 PCR, followed by cleavage with enzyme *RsaI*; lanes 7 and 8: amplification of *E. multilocularis* DNA with Eg16 PCR; lane 9: amplification of *E. granulosus* DNA with Eg9 PCR; lane 10: amplification of *E. granulosus* DNA with Eg9 PCR, followed by cleavage with enzyme *CfoI*; lane 11: amplification of *E. granulosus* DNA with Eg9 PCR, followed by cleavage with enzyme *RsaI*; lane 12: amplification of *E. granulosus* DNA with Eg16 PCR.

All analyzed *E. multilocularis* specimens had identical sequences. The ND1 sequence of *E. multilocularis* from Estonia was submitted to GenBank under accession no. AY855918. The nucleotide sequences obtained were compared with those in the GenBank sequence database. The sequence of the Estonian isolate was identical with other *E. multilocularis* sequences deposited under accession nos. AJ32907, AJ32908, AJ32909, and AJ32910 from Poland (*7*) and AY389984 from China (Yang JK et al., unpub. data), and differed considerably from the sequences of the most closely related species, *E. granulosus*. For phylogenetic analysis, the ND1 sequences of 7 *E. multilocularis*, 24 *E. granulosus*, 1 *Taenia solium*, 1 *E. vogeli*, and 1 *E. oligarthrus* isolates were included and MrBayes 3.04b (*8*) was used for the Bayesian estimation of phylogeny, applying the GTR+I+G substitution model that best fitted the data (determined with Modeltest 3.06) (*9*). Searches were conducted with 4 simultaneous Markov chains over 2 million generations, sampled every 100 generations, and ended with a calculation of a 50% majority rule consensus tree. On the phylogenetic tree, sequences of Estonian isolate group together with those of other *E. multilocularis* isolates from different countries and were clearly separated from those of all other species (data not shown). The results of genetic analysis confirmed morphologic identification of *E. multilocularis*.

This study reports a new location of *E. multilocularis* in Europe. Estonia is the northernmost country on the mainland of the continent where *E. multilocularis* has been described. Because no studies have been published on the occurrence of *E. multilocularis* in Estonia in either foxes or rodents, whether this report identifies a stable endemic area or whether the parasite has expanded its range recently cannot be determined. Although a limited number of foxes were examined, the occurrence of *E. multilocularis* appears to be frequent and widespread in Estonia, which poses a risk for putatively parasite-free adjacent countries in Fennoscandia (*2*).
